# Supplementary material for: LTP induction by structural rather than enzymatic functions of CaMKII
Source: Nature. 2023 Aug 30;621(7977):146–53. doi: 10.1038/s41586-023-06465-y (PMC10482691; doi:10.1038/s41586-023-06465-y)

nature portfolio

https://doi.org/10.1038/s41586-023-06465-y

Supplementary information

LTP induction by structural rather than enzymatic functions of CaMKII

In the format provided by the authors and unedited

SI Guide

LTP induction by structural rather than enzymatic functions of CaMKII

Jonathan E. Tullis[1]*, Matthew E. Larsen[1,2]*, Nicole L. Rumian[1,2], Ronald K. Freund[1], Emma E. Boxer[1,2], Carolyn Nicole Brown[1], Steven J. Coultrap[1], Howard Schulman[3], Jason Aoto[1,2], Mark L. Dell'Acqua[1,2], and K. Ulrich Bayer[1,2‡]

Author affiliations

[1]Department of Pharmacology, and [2]Program in Neuroscience, University of Colorado Anschutz Medical Campus, Aurora, CO 80045, USA

[3]Department of Neurobiology, Stanford University School of Medicine, Stanford, CA94305, USA

*These authors contributed equally to this work.

‡Correspondence should be addressed to K. Ulrich Bayer; email: **ulli.bayer@cuanschutz.edu.**

Table of contents:

SI Figure 1 | Uncropped immunoblots from Main Figures. Proteins were detected by western blotting, using the indicated antibodies and the red rectangles show the cropping location. **a**, Immunoblots for Fig.2b from in vitro kinase assay with increasing concentration of AS283 measuring changes in pT286 and pS831 from purified CaMKII and GST-GluA1. **b**, Immunoblots for Fig.2c showing CaMKII binding to immobilized GST-2BC under ADP or AS283 conditions. **c**, Immunoblots for Fig.3e showing CaMKII binding to immobilized GST-2BC WT under ATP or AS283 conditions. **d**, Immunoblots for Fig.3f showing CaMKII binding to immobilized GST-2BC S1303A under ATP or AS283 conditions. **e**, Immunoblots for Fig.4b showing CaMKII WT, K42M, and F89G phosphorylation of GST-GluA1 at S831.

SI Figure 2 | Uncropped immunoblots from Extended Data Figures. Proteins were detected by western blotting, using the indicated antibodies and the red rectangles show the cropping location. **a**, Immunoblots for ED Fig.1c showing paCaMKII binding to immobilized GST-2BC under dark vs light with and without ADP. **b**, Immunoblots for ED Fig.2b from in vitro kinase assay with increasing concentration of ATP measuring changes in pS831 from purified CaMKII and GST-GluA1. **c**, Immunoblots for ED Fig.2d showing CaMKII binding to immobilized GST-2BC under ADP or AS105 conditions. **d,** Immunoblots for ED Fig.3c showing pT286 in dissociated neurons treated with cLTP or cLTP + AS283. **e**, Immunoblots for ED Fig.3d from in vitro kinase assay measuring pS1303 from purified CaMKII and GST-2BC and inhibition by AS283. **f**, Immunoblots for ED Fig.4a showing CaMKII WT and F89G phosphorylation of pT286 and effect of NM-PP1. **g**, Immunoblots for ED Fig.4c showing CaMKII-F89G binding to immobilized GST-2BC under ADP or NM-PP1 conditions.

Tullis et al., SI Figure 1

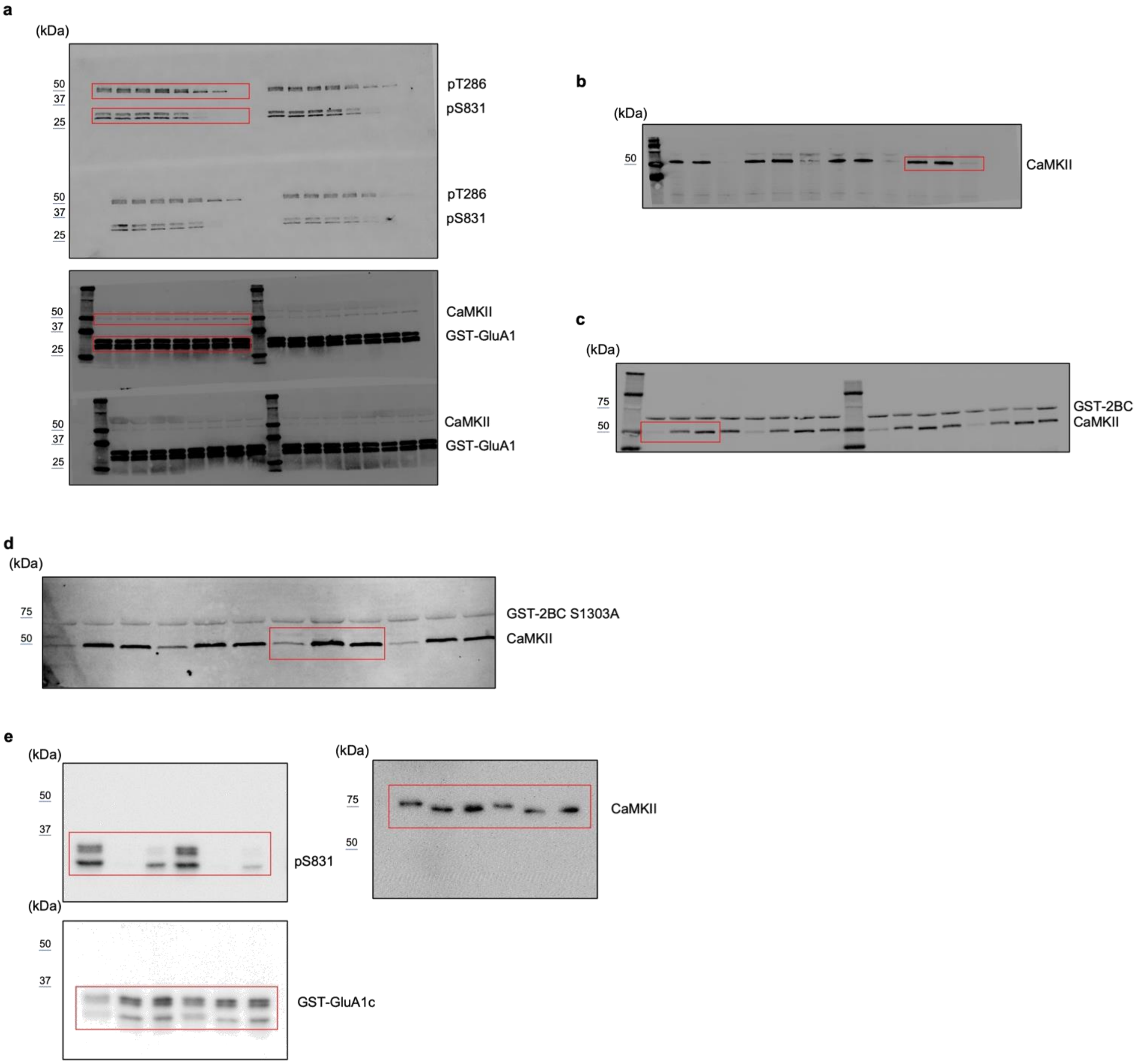

Tullis et al., SI Figure 2

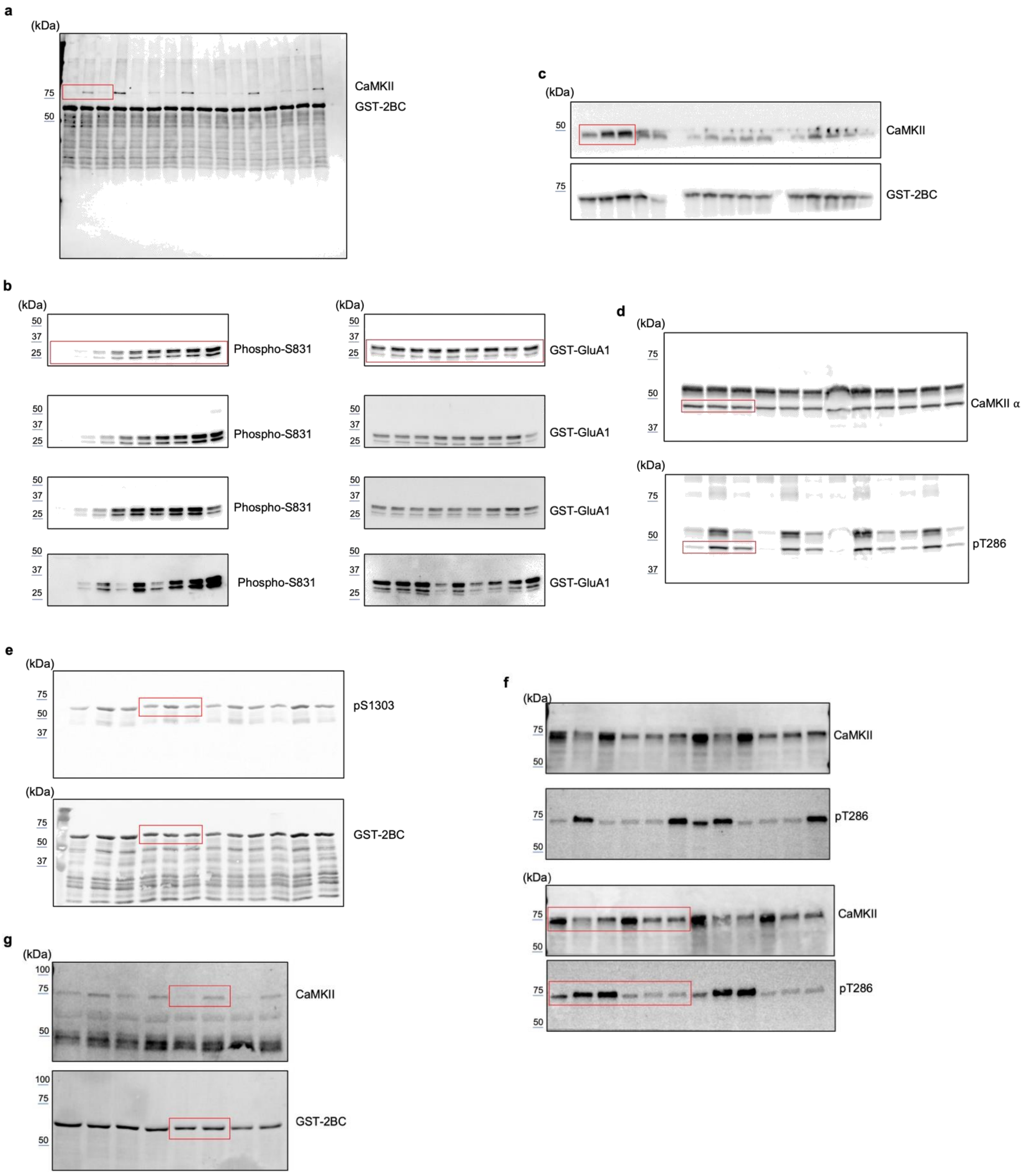

Supplement: Supplementary file 1 — Table of Contents and Supplementary Figs. 1 and 2. [file 41586_2023_6465_MOESM1_ESM.pdf]
