# Supplementary material for: LTP induction by structural rather than enzymatic functions of CaMKII
Source: Nature. 2023 Aug 30;621(7977):146–53. doi: 10.1038/s41586-023-06465-y (PMC10482691; doi:10.1038/s41586-023-06465-y)
Supplement: Supplementary file 2 — Reporting Summary [file 41586_2023_6465_MOESM2_ESM.pdf]

nature portfolio

Corresponding author(s): *Double-blind peer review submissions: write DBPR and your manuscript number here instead of author names.*

Last updated by author(s): *YYYY-MM-DD*

Reporting Summary

Nature Portfolio wishes to improve the reproducibility of the work that we publish. This form provides structure for consistency and transparency in reporting. For further information on Nature Portfolio policies, see our Editorial Policies and the Editorial Policy Checklist.

Statistics

For all statistical analyses, confirm that the following items are present in the figure legend, table legend, main text, or Methods section.

n/a	Confirmed	
☐	☒	The exact sample size (*n*) for each experimental group/condition, given as a discrete number and unit of measurement
☐	☒	A statement on whether measurements were taken from distinct samples or whether the same sample was measured repeatedly
☐	☒	The statistical test(s) used AND whether they are one- or two-sided<br>*Only common tests should be described solely by name; describe more complex techniques in the Methods section.*
☐	☒	A description of all covariates tested
☐	☒	A description of any assumptions or corrections, such as tests of normality and adjustment for multiple comparisons
☐	☒	A full description of the statistical parameters including central tendency (e.g. means) or other basic estimates (e.g. regression coefficient) AND variation (e.g. standard deviation) or associated estimates of uncertainty (e.g. confidence intervals)
☐	☒	For null hypothesis testing, the test statistic (e.g. *F*, *t*, *r*) with confidence intervals, effect sizes, degrees of freedom and *P* value noted<br>*Give P values as exact values whenever suitable.*
☒	☐	For Bayesian analysis, information on the choice of priors and Markov chain Monte Carlo settings
☒	☐	For hierarchical and complex designs, identification of the appropriate level for tests and full reporting of outcomes
☐	☒	Estimates of effect sizes (e.g. Cohen's *d*, Pearson's *r*), indicating how they were calculated

Our web collection on statistics for biologists contains articles on many of the points above.

Software and code

Policy information about availability of computer code

Data collection	Slidebook (Intelligent Imaging Innovations [3i], Version 6.0)
Data analysis	ImageJ (Version: 2.9.0/1.53t)

For manuscripts utilizing custom algorithms or software that are central to the research but not yet described in published literature, software must be made available to editors and reviewers. We strongly encourage code deposition in a community repository (e.g. GitHub). See the Nature Portfolio guidelines for submitting code & software for further information.

Data

Policy information about availability of data

All manuscripts must include a data availability statement. This statement should provide the following information, where applicable:

- Accession codes, unique identifiers, or web links for publicly available datasets
- A description of any restrictions on data availability
- For clinical datasets or third party data, please ensure that the statement adheres to our policy

doi: 10.17632/dbn4fv37xy.1

Human research participants

Policy information about studies involving human research participants and Sex and Gender in Research.

Reporting on sex and gender	N/A
Population characteristics	N/A
Recruitment	N/A
Ethics oversight	N/A

Note that full information on the approval of the study protocol must also be provided in the manuscript.

Field-specific reporting

Please select the one below that is the best fit for your research. If you are not sure, read the appropriate sections before making your selection.

☒ Life sciences ☐ Behavioural & social sciences ☐ Ecological, evolutionary & environmental sciences

For a reference copy of the document with all sections, see nature.com/documents/nr-reporting-summary-flat.pdf

Life sciences study design

All studies must disclose on these points even when the disclosure is negative.

Sample size	Sample size was determined with a power analysis based off the preliminary data effect size and variability
Data exclusions	No data exclusions
Replication	Biological replication was achieved by measuring each unique cell, sample, and hippocampal slice once, derived from at least two separate cultures and a minimum of four distinct wells. Sample size was at minimum 3 independent samples.
Randomization	Randomization was accomplished by reversing the sample order for every experiment
Blinding	Investigators were not blinded to the samples during collection or analysis. Blinding was not performed due to resources constraints combined with the nature of experiments and analysis having low potential for introducing bias

Reporting for specific materials, systems and methods

We require information from authors about some types of materials, experimental systems and methods used in many studies. Here, indicate whether each material, system or method listed is relevant to your study. If you are not sure if a list item applies to your research, read the appropriate section before selecting a response.

Materials & experimental systems

n/a	Involved in the study	
☐	☒	Antibodies
☐	☒	Eukaryotic cell lines
☒	☐	Palaeontology and archaeology
☐	☒	Animals and other organisms
☒	☐	Clinical data
☒	☐	Dual use research of concern

Methods

n/a	Involved in the study	
☒	☐	ChIP-seq
☒	☐	Flow cytometry
☒	☐	MRI-based neuroimaging

Antibodies

Antibodies used	CB 2, available at Invitrogen but made in house; Invitrogen cat# 13-730-0.<br>anti-CaMKII (1:2000, BD); BD Transduction Laboratories; #611293; lot# 99135.<br>pT286-CaMKII (1:2500, Phospho-Solutions); #p1005-2886; lot# ks921b.<br>anti-GST (1:2000, Millipore); #AB3282; lot# 3083109<br>pS831-GluA1 (1:2000, Phospho-Solutions); #p1160-831; lot# cs921p.<br>pS1303-GluN2B (1:2000, Millipore); #07-398; lot# 3792158.

	goat-anti mouse (1:10,000 GE); Sheep anti-mouse-HRP; #NA931V; lot# 17205275<br>goat-anti rabbit (1:10,000 GE); Donkey Anti-Rabbit-HRP; #NA934V; lot# 17469003<br>CyDye 700 goat anti mouse (1:10,000 Cytiva); #29360784, lot# OF29A-XP<br>Cydye 800 goat-anti rabbit (1:10,000 Cytiva); #29360790; lot# OF-29C-XP
Validation	Antibodies for CaMKII and pT286 were validated using KO or KI (T286A) samples in ref #51.<br>anti-GST antibody was validated at Millipore AB3282; RRID: AB_91439<br>anti-pS831-GluA1 antibody was validated at Phospho-Solutions and by in vitro phosphorylation in ref #51<br>anti-pS1303-GluN2B (aka NR2B) was validated at Millipore cat. # 07-398 and by in vitro phosphoryation in ref #15

Eukaryotic cell lines

Policy information about cell lines and Sex and Gender in Research

Cell line source(s)	HEK-293 cells
Authentication	Were not authenticated
Mycoplasma contamination	Were not tested for mycoplasma
Commonly misidentified lines (See ICLAC register)	No commonly misidentified lines were used in this study (according to ICLAC register)

Animals and other research organisms

Policy information about studies involving animals; ARRIVE guidelines recommended for reporting animal research, and Sex and Gender in Research

Laboratory animals	Mice; C57BL/6; 8-10 weeks. Wildtype, CaMKII KO, CaMKII T286A, GluN2B KI
Wild animals	N/A
Reporting on sex	Findings of WT and T286A KI animals apply to males. Findings of AAV-injected CaMKII KO apply to both males and females.
Field-collected samples	N/A
Ethics oversight	University of Colorado Institutional Animal Care and Use Committee (IACUC)

Note that full information on the approval of the study protocol must also be provided in the manuscript.
